# Exploring machine learning algorithms to predict acute respiratory tract infection and identify its determinants among children under five in Sub-Saharan Africa

**DOI:** 10.3389/fped.2024.1388820

**Published:** 2024-11-20

**Authors:** Tirualem Zeleke Yehuala, Bezawit Melak Fente, Sisay Maru Wubante, Nebiyu Mekonnen Derseh

**Affiliations:** ^1^ Department Health Informatics, Institute of Public Health, College of Medicine and Health Sciences, University of Gondar, Gondar, Ethiopia; ^2^ Department of General Midwifery, School of Midwifery, College of Medicine and Health Sciences, University of Gondar, Gondar, Ethiopia; ^3^ Department of Epidemiology and Biostatistics, Institute of Public Health, College of Medicine and Health Sciences, University of Gondar, Gondar, Ethiopia

**Keywords:** prediction, acute respiratory infection, machine learning, Sub-Saharan Africa, SHAP (SHapley additive exPlanations), hyper parameter tuning

## Abstract

**Background:**

The primary cause of death for children under the age of five is acute respiratory infections (ARI). Early predicting acute respiratory tract infections (ARI) and identifying their predictors using supervised machine learning algorithms is the most effective way to save the lives of millions of children. Hence, this study aimed to predict acute respiratory tract infections (ARI) and identify their determinants using the current state-of-the-art machine learning models.

**Methods:**

We used the most recent demographic and health survey (DHS) dataset from 36 Sub-Saharan African countries collected between 2005 and 2022. Python software was used for data processing and machine learning model building. We employed five machine learning algorithms, such as Random Forest, Decision Tree (DT), XGBoost, Logistic Regression (LR), and Naive Bayes, to analyze risk factors associated with ARI and predict ARI in children. We evaluated the predictive models’ performance using performance assessment criteria such as accuracy, precision, recall, and the AUC curve.

**Result:**

In this study, 75,827 children under five were used in the final analysis. Among the proposed machine learning models, random forest performed best overall in the proposed classifier, with an accuracy of 96.40%, precision of 87.9%, F-measure of 82.8%, ROC curve of 94%, and recall of 78%. Naïve Bayes accuracy has also achieved the least classification with accuracy (87.53%), precision (67%), F-score (48%), ROC curve (82%), and recall (53%). The most significant determinants of preventing acute respiratory tract infection among under five children were having been breastfed, having ever been vaccinated, having media exposure, having no diarrhea in the last two weeks, and giving birth in a health facility. These were associated positively with the outcome variable.

**Conclusion:**

According to this study, children who didn't take vaccinations had weakened immune systems and were highly affected by ARIs in Sub-Saharan Africa. The random forest machine learning model provides greater predictive power for estimating acute respiratory infections and identifying risk factors. This leads to a recommendation for policy direction to reduce infant mortality in Sub-Saharan Africa.

## Introduction

Children's immune systems are particularly vulnerable to infection (1). In developing nations, malnutrition, diarrheal diseases, and acute respiratory infections (ARI) are the main causes of illness and mortality among children (2). One of the most prevalent diseases in children is acute respiratory tract infection, which nearly always causes serious health issues and even leads to death in children under five (1). The most common acute respiratory infections are influenza, common cold, sinusitis, tonsillitis, and laryngitis. They are caused by viruses. It is the greatest global public health burden, and developing nations particularly those in Sub-Saharan Africa (SSA), where Ethiopia is located continue to bear an excessive burden of this infection (2). Approximately 20% of the deaths of children globally are caused by acute respiratory infections (ARI) (3). With 80% occurring in Sub-Saharan Africa and southern Asia in 2021 (4). In Sub-Saharan Africa, the highest child mortality rate was 27 deaths per 1,000 live births. Children born in Sub-Saharan Africa are 11 times more likely to die in the first month of life than those born in Australia and New Zealand (5). Similar to this, numerous poor wealth statuses have been attributed to acute tract infections in Sub-Saharan African countries (2, 6, 7). ARI diseases rank fourth among childhood illnesses with a higher rate of morbidity, according to WHO data from 2019. ARI illnesses rank higher among communicable diseases that cause death than other comorbidities when compared with malaria (8). Some previous studies found wealth status, breastfed, place of delivery, birth size*,* media exposure, diarrhea, stunting, and wasting were the most significant predictors of ARI (3, 4). Despite the fact that malnutrition and an appropriate low birth weight are linked to a very high risk of dying from ARI in developing countries. Even though various local studies on the prevalence and factors associated with ARIs among children under the age of five have been conducted in Sub-Saharan African countries. Used a variety of models and methodologies using conventional methods, including retrospective analysis, inferential statistics, survival analysis, regression models, mapping and spatial analysis, multilevel analysis, and multivariate decomposition (4–7). Recent research suggested machine learning, data mining, and deep learning (DL) have the potential to speed up this progress. These techniques have shown significant performance in a variety of fields, including the fields of public health and medicine. Nevertheless, there is not enough proof to support the prediction of ARIs and the identification of determinants using machine learning techniques, and no prior research has attempted to do so in Sub-Saharan African countries. A predictive model enables real-time children's’ ARI of risk stratification, which guides primary attention to care for the children's good health outcomes. The decision-making process is automated using machine learning algorithms. When these methods are utilized in the healthcare sector, patients’ health outcomes are enhanced and healthcare costs are decreased. We create a novel method for predicting them using machine learning algorithms that determine the child's ARI status.

This study made use of data from the Demographic and Health Surveys (DHS), which were carried out using nationally representative samples in 36 Sub-Saharan African nations between 2005 and 2019.Hence, this study aimed to predict ARI status and identify its predictors using the current state-of-the-art machine learning models.

In conclusion, the two primary questions this study seeks to address are as follows:

### RQ1

Which determinants are the most significant for acute respiratory infection (ARI) status?

### RQ2

Which machine learning models help to effectively predict acute respiratory infection (ARI) status?

## Methods

### Study setting

This study was conducted in Sub-Saharan African countries using the most recent Demographic and Health Survey (DHS) dataset from 36 Sub-Saharan African countries. Geographically, east Africa is a sub-region of Africa that includes 54 internationally known countries, among which Angola, Burkina Faso, Benin, Burundi, Dr Congo, Congo, Cote devoirs, Cameron, Ethiopia, Gabon, Ghana, Gambia, Guinea, Kenya, Comoros, Liberia, Lesotho, Madagascar, Mali, Malawi, Mozambique, Nigeria, Niger, Namibia, Rwanda, Sera Leone, Senegal, Sao Tome, Swaziland, Chad, Togo, Tanzania, Uganda, South Africa, Zambia, and Zimbabwe conducted the study.

### Data source

This study used measured DHS program data, accessed online, comprising the Kids Record dataset (KR file). Among 54 Sub-Saharan African countries, 36 DHS datasets were eligible for analysis since the rest of the Sub-Saharan African countries had no recorded acute respiratory infection, and we did not get the DHS dataset in the years from 2005 to 2022 that were included in this study.

### Sample size determination and sampling technique

The DHSs were a nationally representative survey that collected data on basic health indicators like mortality, morbidity, family planning service utilization, fertility, and maternal and child health-related indicators (8). This study used a weighted sample of 75,827 children aged under five across 36 Sub-Saharan African countries using the recent DHS dataset. For this study, we used the Kids Record dataset (KRFile). A two-stage stratified cluster sampling technique was used to select study participants. In the first step, a stratified sample of enumeration areas (EAs) was selected at random; in the second stage, households were selected using systematic random sampling in the selected EAs. In each selected household, mother or father were interviewed with an individual questionnaire.

### Population, and eligibility criteria

In this study, only children under age five who had symptoms of acute respiratory infection (ARI) in the two weeks before the survey in Sub-Saharan African during the survey period who were in the selected enumeration areas at the time of DHS data collection were the study populations and included in this study.

### Study design and study period

This study adopted a design science approach for analysis and building a model of the DHS dataset, which was conducted from 2005 to 2022. The design science approach focuses on solving practical problems through the creation and evaluation of innovative artifacts. Through this approach, the study contributes to both theoretical and practical aspects by offering a novel solution to a specific problem (9). Finally, we develop a predictive model that predicts the determinants or factors of acute respiratory infection among under-five children.

### Study variables

#### The outcome variables

Acute respiratory tract infection is defined as children having a history of coughing within two weeks preceding the survey. Children under age five who had symptoms of acute respiratory infection (ARI) in the two weeks before the survey. Then, the outcome variable was categorized as yes or no (acute respiratory infection). The first category [children under age five who had symptoms of acute respiratory infection (ARI) in the two weeks] was given a “1” value, and the second category (children who had no symptoms of acute respiratory infection) was given a value of “0,” respectively. This classification and analysis was conducted according to the guide to the DHS (10, 11).

#### Independent variables

The features (independent variables) used in this study include individual, household, community, and health service factors. Incorporate the mother's age (15–24, 25–34, and 35–49), birth size (small, normal, large), stunting (normal or severe), underweight (normal or severe), media exposure (yes or no), breastfeeding (ever or never), diarrhea (yes or no), and the child's vaccination history (yes or no). And Sub-Saharan countries (central, east). The health service factors determine the mode of delivery of services (health facility, home).

### Data analysis procedure

**Figure 1:**
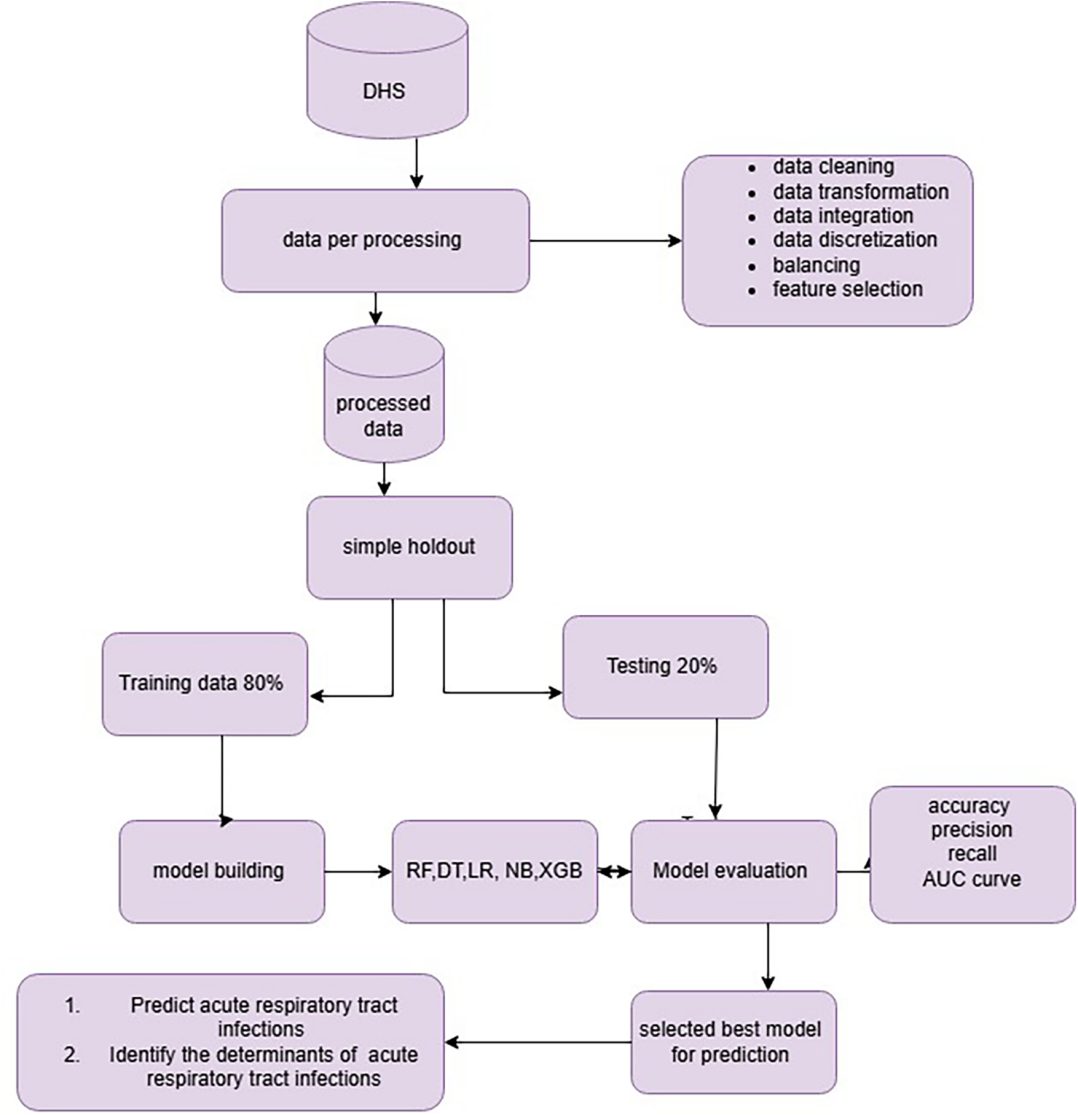
Overall data preparation and analysis process for predicting acute respirator trace infection.

Machine learning algorithms were used to come up with objective predictions about acute respiratory tract infection and to identify the factors that influence respiratory tract infection. Data processing is a machine learning technique that transforms raw data into an understandable format (12). Data processing and analysis will be performed using Python software and some basic packages like Panda, Scikit-Learn, Imblearn, Numpy, and Seaborne utilized for gathering data, preparing data, discretizing data, transforming data, and choosing, training, and evaluating models. Finally, we develop a predictive model that predicts both the acute respiratory tract infection and its associated determinants (Figure 1).

### Data preprocessing

We employed data preprocessing to remove incomplete values, noisy data, outliers, and incompatible data since it is done in its raw form. Major data Pre-processing, such as data cleaning, data integration, transforming, and discretizing data, is important (13). In this study, we utilized major t data preprocessing.

### Data cleaning

Data cleaning is the most important process for data analysis to ensure the dataset removes incorrect or erroneous data (14). We employed data cleaning, which typically includes noise and missing values. Raw data is always missing, and that data cannot be sent through modeling, building, or testing (15). We utilized data cleaning, which typically includes outlier values, imbalanced outcome variables, noise, and missing values. A large fraction of the 75,827 records available had less than 579 missing data for the critical 16 features, comprising nearly 1.7% of the data set. Missing values are present in our dataset for both continuous and categorical data. For the continuous and categorical variables, we addressed these missing values using mean and mode imputation, respectively. We found outliers in the continuous nature of the data and dealt with them using visualization methods like the box-plot and subplot.

### Feature selection

Feature selection is a process of removing irrelevant or redundant features from the number of features while developing a predictive model (16). In this study, the dataset includes over a thousand features; therefore, feature selection is essential. Excessive features make it time-consuming and resource-intensive (17). The feature selection methods were used in data preprocessing to achieve efficient data reduction and select the most important determinants. In this study, we utilized recursive feature elimination (RFE) and SHAP values to identify the most relevant variables for predicting acute respiratory infection among under-five-year-old children.

### Data transformation

Data transformation involves converting the data into one format that is suitable for analysis, which involves converting data types, scaling data, normalizing data, smoothing, and renaming (18). In this study, we employed one hot-encoding technique to convert string data to an integer to provide the uniform data type for machine learning classifiers. Before creating the model, we also scaled the dataset to make it look uniform, suitable for analysis and improving model training and evaluation.

### Data discretization

In order to make the data easier to analyze, we did data discretization, which changes continuous data in the interval or range. In this study, we use binning as a method for converting continuous input into categorical input in order to improve the performance and interpretability of classification algorithms. By labeling continuous input into distinct groups or bins, the algorithm is able to make distinctions between different classes of data based on specific values of the input variables. Mother's age is continuous; variables were discretized into 15–24, 25–34, and 35–49 according to DHS guidelines.

### Data splitting

In this study, we utilized a simple holdout method like 80% for training and 20% for testing to ensure robust model evaluation. The dataset was divided into training and testing sets, with the former used to build and train the model and the latter used to evaluate its performance on previously unseen data.

### Class balancing

Before training the prediction model, an unbalanced dataset was resampled; this might be viewed as a data pretreatment step. Machine learning algorithms are prone to bias toward the majority class when given an unbalanced data set (19, 20). To avoid machine learning models biased toward the majority class (acute respiratory infection), the dataset was balanced using the synthetic minority oversampling technique. We employed SMOTE oversampling by creating synthetic examples (new observations) that resemble the minority class by interpolating between minority classes samples in the feature space rather than creating exact copies of existing examples. SMOTE has been shown to almost continually increase classification performance for resampling imbalanced datasets (21).

### Model selection

The predicted variable in this study was binary classification, since acute respiratory infection status was divided into two “yes” or “no.” For model building, five classifiers: random forests, XGBoost, logistic regression, Naïve Bayes, and decision trees were used. The algorithms were chosen in accordance with previous research that used machine-learning methods to predict tasks (22, 23). The rationale behind its ease of implementation, interpretability, training efficiency, reduction of overfitting, and speed in predicting unknown records (24, 25). Our aim for this study is to apply the ML method to predict acute respiratory tract infections and to provide insight for the government and policymakers.

### Decision tree

In this study is a novel integrated supervised learning algorithm to efficiently handle vast amounts of survey data. As a result, the study's technique is novel and innovative, combining theory and practice due to its predictability and ease of use. Decision trees are one of the most popular approaches for representing classification (26). Due to easily interpretable machine learning algorithms, they may be pretty powerful when used in ensemble algorithms and robust to outliers.

### Random forest

Random forest is a machine learning algorithm that ensembles multiple decision trees to make predictions for classification and regression problems. The concept of multiple random tree generation is used in each split decision, along with a voting system, sample bagging, training bootstrapping, and randomly selected features. Random Forest overcomes these limitations of decision trees by using an ensemble of decision trees. An ensemble of models is used by the machine learning process known as bootstrap aggregating, sometimes known as bagging, to increase prediction accuracy and stability (27).

### XGBoost (extreme gradient boosting)

An ensemble machine learning algorithm based on decision trees is used in Extreme Gradient Boosting, or XG Boost, a supervised learning technique (28). Each independent variable is given a weight, which the decision tree then uses to generate predictions. The Extreme Gradient Boosting classifier is an adaptable technique that combines numerical and categorical features in an easy-to-read format. It can handle the overfitting problem, but due to its sensitivity to outliers, scalability on bigger datasets is a concern.

### Evaluation criteria

In this study, the performances of predictive models were evaluated. We divided the dataset into training (80%) and test (20%) sets. Then, the performance of the trained models was evaluated using the test set based on the criteria of accuracy score, ROC curve, and precision (P), recall (R), and F-measure as follows:

$$\begin{array}{rl} & \mathrm{Precision}=(\mathrm{TP})/(\mathrm{TP}+\mathrm{FP})\\ & \mathrm{Recall}=(\mathrm{TP})/(\mathrm{TP}+\mathrm{FN})\\ & F\text{–}\mathrm{Measur}=(2*\mathrm{Precisio}*\mathrm{Recall})/(\mathrm{Precision}+\mathrm{Recall})\\ & \mathrm{Accuracy}=((\mathrm{TP}+\mathrm{TN})/(\mathrm{TP}+\mathrm{TN}+\mathrm{FP}+\mathrm{FN}))\times 100 \end{array}$$

In Table 1 a false positive (FP) indicates not symptoms of acute respiratory tract infection that were incorrectly identified as having symptoms of acute respiratory tract infection; a true positive (TP) indicates had symptoms of acute respiratory tract infection that were correctly identified as having symptoms of acute respiratory tract infection; a true negative (TN) indicates not symptoms of acute respiratory tract infection correctly identified as not having symptoms of acute respiratory tract infection; and a false negative (FN) indicates had symptoms of acute respiratory tract infection incorrectly identified as not having symptoms of acute respiratory tract infection (29). Furthermore, the ROC curve (receiver operating characteristics curve) provides a comprehensive assessment of the accuracy of a model by screening the range of threshold values for the decision-making.

**Table 1:** Confusion matrix.

*N* = Number of instances		Confirmed by observation	
Predicted by test		Yes	No
	Yes	TP (had symptoms ARI)	FP ()
	No	FN ()	TN(had no symptoms ARI)

### Hyper parameter tuning

In this study, we utilized hyper parameter tuning for selecting the optimal values for the machine learning model. Grid search was used to adjust the selected algorithm's hyper parameters since choosing the right hyper parameter has always been a critical stage in the creation of machine learning models and greatly affects model prediction performance (30, 31).

### Results sociodemographic characteristics of the study participant

In this study, we investigated a sample of 75,827 children under the age of five from 36 countries in Sub-Saharan Africa that were part of a demographic and health survey. Overall, it was shown that 11% of children with symptoms of acute respiratory tract infection (ARI) disease and 89% of children who had no symptoms of ARI disease. Demonstrates that compared to vaccinated children (8%), children who were not vaccinated had a higher prevalence of acute respiratory tract infections (53%), and approximately 6% of children with ARI symptoms were never breastfed, while 82.3% of children who were properly breastfed did not exhibit any ARI symptoms. Approximately 30% of children were delivered at home with ARI symptoms, compared to 6% who were delivered in a medical facility. Compared to the 6.3% of children who experienced media exposure and exhibited ARI symptoms, 48.7% of children did not exhibit any symptoms. The majority of children reported were found to have stunting status; 51% were in the severe stage. Among those, 46% had ARI symptoms, compared to 5% who had no ARI symptoms. The results of this study revealed that there is a high ARI symptom rate seen in central Sub-Saharan Africa (6%) and east Sub-Saharan Africa (5.5%) this present (Table 2).

**Table 2:** Description of acute respiratory tract infection in Sub-Saharan Africa countries, evidences from DHS (*N* = 75,827).

Variable	Frequency (%)	ARI	
		YES	NO
Ever vaccinated			
Yes	46,338 (61%)	6,704 (8%)	39,634 (53)
No	29,018 (39%)	27,191 (36%)	1,827 (3%)
Breastfed			
Ever	70,154 (92%)	7,411 (9.7)	62,743 (82.3)
Never	5,673 (8%)	4,553 (6%)	1,120 (2%)
Diarrhea			
Yes	15,363 (20)	2,724 (3.6)	12,639 (17.4)
No	60,464 (80)	5,807 (7.7)	54,657 (72.3)
Place delivery			
Health facility	50,014 (65%)	5,261 (6%)	44,753 (59%)
Home	25,813 (34%)	22,543 (30%)	3,270 (4)
Stunting			
Normal	27,711 (36.5)	3,584 (4.7%)	24,127 (31.8%)
Moderate	9,229 (12.5)	1,071 (1.8%)	8,158 (10.7)
Severe	38,887 (51%)	35,011 (46%)	3,876 (5%)
Media exposure			
Yes	41,618 (55%)	4,842 (6.3%)	36,776 (48.7%)
No	34,209 (45%)	3,689 (4.8%)	30,520 (40.2%)
Birth size			
Normal	33,179 (44%)	2,906 (4%)	30,273 (40%)
Large	26,032 (34%)	3,175 (4%)	22,857 (30%)
Small	16,616 (22%)	14,166 (18.8)	2,450 (3.2)

### Class balancing

In order to balance the target features for this study, we applied the Synthetic Minority Oversampling Technique (SMOTE). This technique generates additional synthetic observations from the minority category in order to balance the unequal distribution of the outcome variable. Prior to smote balancing, the prevalence of children not having symptoms of ARI was 67,483 (89%), while the prevalence of children having symptoms of ARI was 8,341 (11%). We obtained a balanced sample of children who had ARI with counts of label 67,483 and children who had ARI during with counts of label 67,483.

### Feature selection

**Figure 2:**
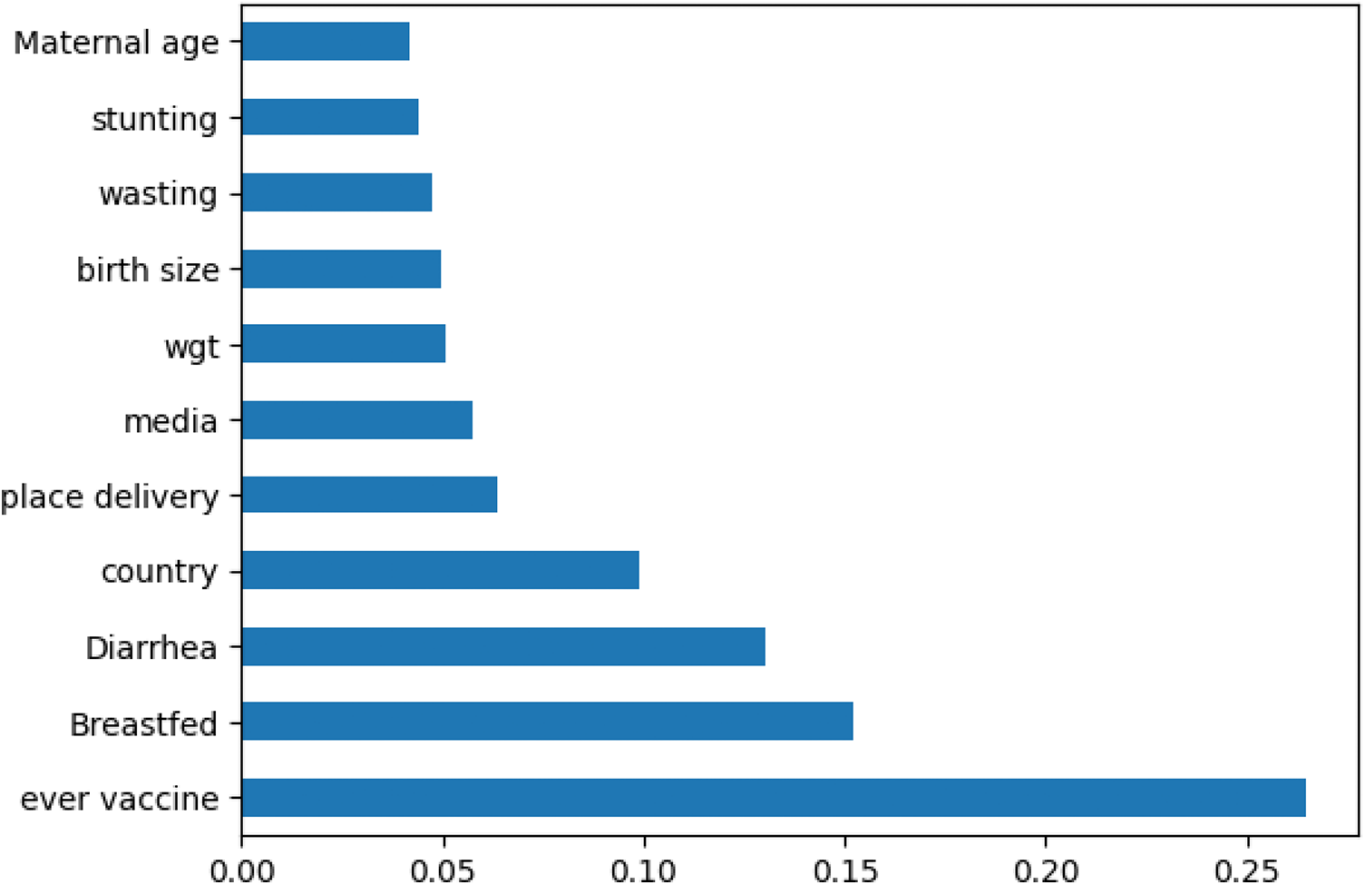
Importance features selected by recursive feature selection.

In this study, the recursive feature selection method (RFE) was utilized. Feature selection and feature importance rank were techniques for identifying a subset of features by removing irrelevant or redundant features. The importance of feature selection was reducing the cost of learning by reducing the number of features, increasing model performance, and reducing storage and time. In this study, the dataset contains 45 features with 75,827 records. To select the most important features, we employed fast recursive feature elimination. This approach offers flexibility in controlling the number of features retained and infers features’ relevance using an estimate of their importance from the algorithm; all features are selected. The most important features—mother age, breastfed, place of delivery, birth size*,* media exposure, diarrhea, stunting, underweight, country, wasting, ever vaccinated, and weight were selected by (fast recursive feature) RFE, and these determinants were used for model building Figure 2.

### Model explanation using SHAP values

The high values of the ever vaccine variable have a high negative contribution to the ARI, while low values have a high positive contribution (32). Interpreting the results of machine learning algorithms can be significantly more challenging compared to classical statistical analysis methods. It is challenging to interpret how predictions were made, but techniques like SHAP provide a unified framework, proposed by Lundberg and Lee, to interpret the outputs of a wide range of machine learning models by calculating SHAP values to gain insights into the contributions of individual determinants for the model's predictions.

**Figure 3:**
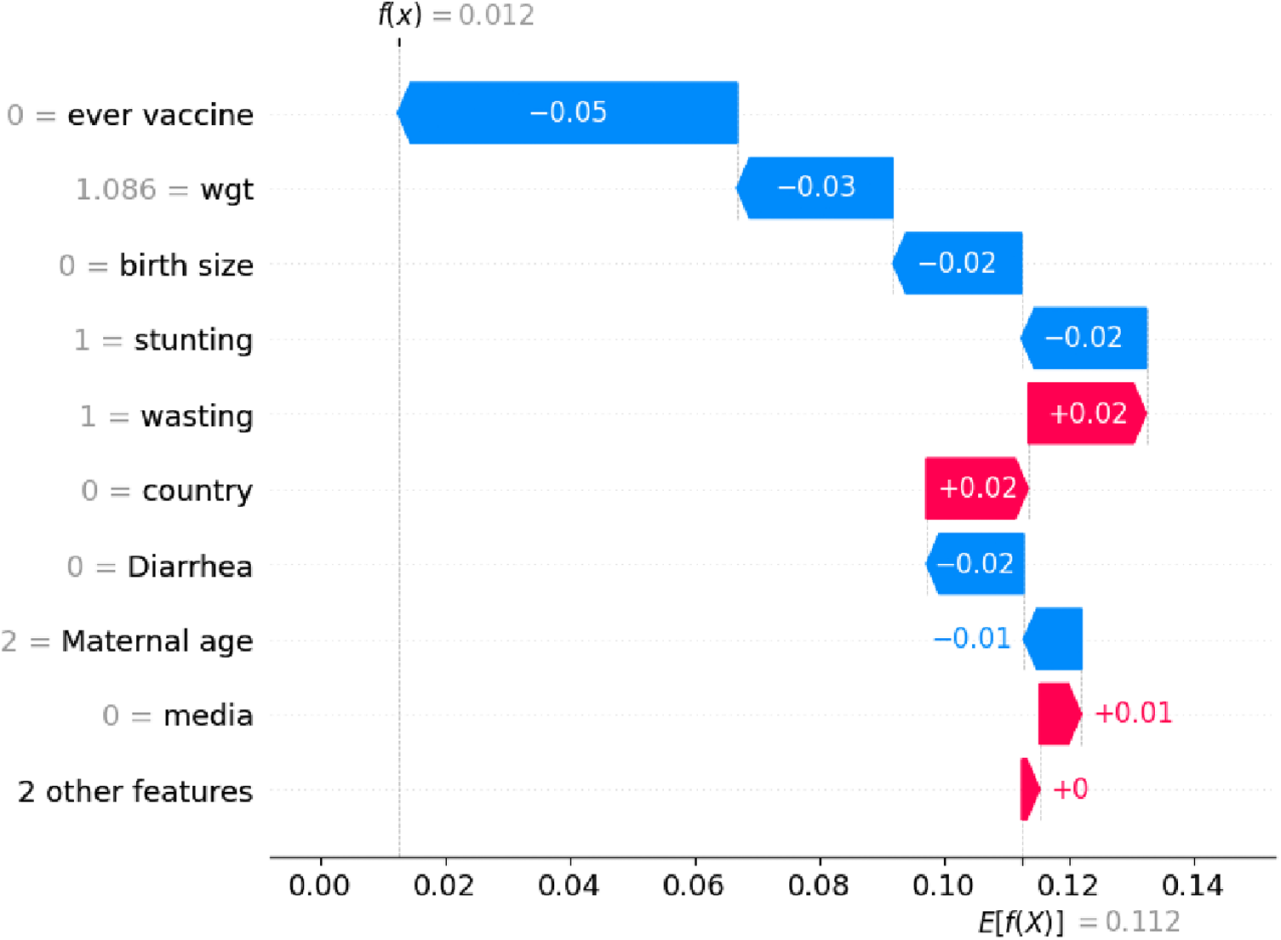
The positive (in red) and negative (in blue) contributions features for acute respirator trace infection among under five children.

In this study, we employed the Random Forest Classifier in combination with model-agnostic SHAP values to find the most significant predictors of acute respiratory infections. By evaluating the mean absolute SHAP values throughout the dataset, the study identified the most important predictors of acute respiratory infections. As shown in (Figure 3), where the SHAP values are positive, features contribute to an increased children having no symptom ARI. This is represented by the red line, indicating the category coded as “1” or a high value, and SHAP values are negative; this is represented by the blue line, indicating the category coded as “0” (had symptom of ARI) or a low value. The features tend to increase the predicted values of children who had no symptom ARI, such as features like having media exposure, being ever vaccinated, giving birth in a health facility, having no diarrhea in the last two weeks, and the stunting status. Other features have a positive impact; children had not acute respiratory infections. The features like not ever vaccinated, the stunting status being severe, having diarrhea in the last two weeks, having no media exposure, and giving birth at home have a negative impact on children with ARI.

### Training and testing data

The data set was divided into training and test sets. Eighty percent (80%) of the input dataset is used for model building, while the remaining twenty percent (20%) is used for validating the model. which are 60,661 samples and 15,166 samples for training and testing, respectively. In all experiments, 11 selected attributes (ever vaccinated, breastfed, place of delivery, birth size*,* media exposure, diarrhea, stunting, country, wasting, and weight) were used. The outcome variable is a binary response, which is acute respiratory infection was “yes” or “not.”

### Comparisons of selected machine learning model

The goal of this study was to build a predictive model for acute respiratory infection and identify the important determining children who had acute respiratory symptoms for evidence-based decision-making. Supervised machine learning algorithms were used, such as Random Forest, Decision Tree (DT), Logistic Regression (LR), XGB (Extreme Gradient Boosting), and the Naïve Bayes method, with the same testing parameters. Since accuracy, AUC, precision, recall, and F-measure are the parameters used to evaluate the performance of the model.

After comparing proposed machine learning models, random forest emerged as the best predictive model with an accuracy of 96.40%, precision of 87.9%, F-measure of 82.8%, ROC curve of 94%, and recall of 78%, with parameters criterion=’entropy’,max_features=’sqrt’, min_samples_split = 13, estimators = 500, random state = 0, max_depth = 22, max_leaf_nodes = 500, jobs = −1, random forest is the best classifier in this study. In addition, random forests had high specificity (98%) and sensitivity (78%).

The true positive rate of random forest was 87%, the false positive rate was 2.6%, and the AUC curve was high, 94%. Moreover, decision tree (DT) accuracy of 92.8%, recall of 98%, precision of 93%, F1 score of 96%, and an average AUC curve of 84% with parameters criterion=’entropy’,max_features=’sqrt’,min_samples_split = 12, random_state = 0, max_depth = 30, max_leaf_nodes = 600.

**Figure 4:**
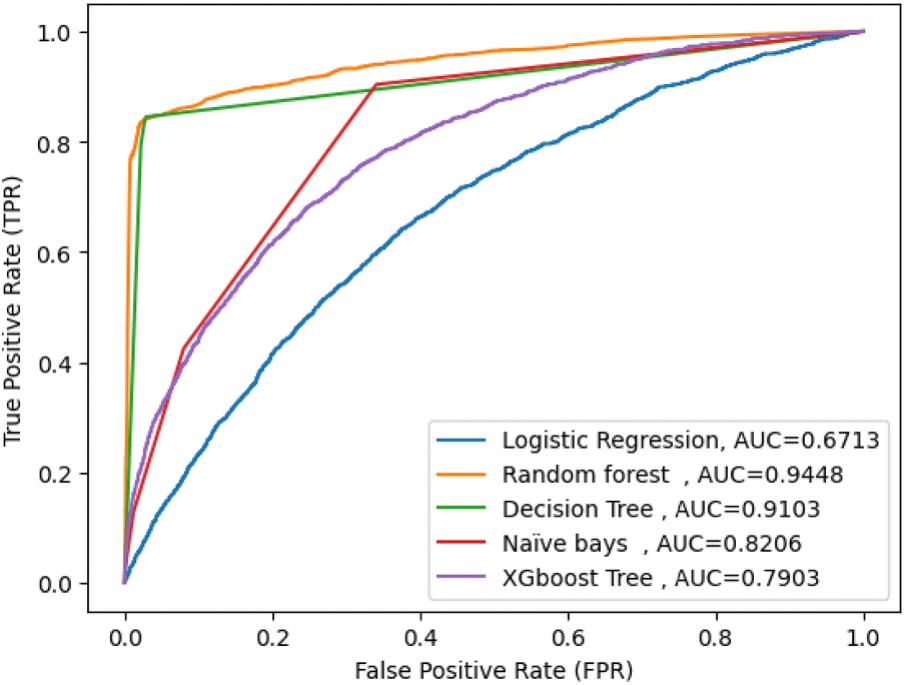
AUC score for five machine learning model.

The decision tree had high specificity (97%) and sensitivity (79%). The true positive rate of the decision tree was 87%, the false positive rate was 2.8% among the proposed models, and Naïve Bayes was the one with an accuracy of 87.55%, precision of 67%, F-measure of 48%, recall of 53%, and an AUC score of 82%. The low true positive rate was 4.4% and the high false positive rate was 98%; hence, the Naïve Bayes model was highly misperdicted, as shown in (Figure 4 and Table 3).

**Table 3:** Accuracy, precision, recall and F-measure for the machine learning algorithms.

ML model	Accuracy	Precision	Recall	F-measure	AUC	Parameters
Random forest(RF)	96.40%	87.9	78%	82.8	94	criterion=’entropy’, max_features=’sqrt’, min_samples_ split = 13, estimators = 500, random state = 0, max_depth = 22, max_leaf_nodes = 500, jobs = −1
Decision Tree (DT)	95.76%	82	79	80	91	criterion=’entropy’,max_features=’sqrt’, min_samples_split = 12, random state = 0, max_ depth = 30, max_leaf_nodes = 600
XGB(Extreme Gradient Boosting)	89.30%	86	42	60	80	criterion=’entropy’,max_features=’sqrt’, min_samples_ split = 13, estimators = 500, random state = 45, max_depth = 32, max_leaf_nodes = 100
Logistic Regression (LR)	88.90%	88%	57	64	67	max_depth = 2, learning rate = 0.2, estimators = 100,silent = True, objective=’binary: logistic’ booster=’gbtree’, jobs = 1, nthread = None
Naïve Bayes	87.53%	67	53	48	82	depth = 3, iterations = 202, l2_ leapfrog = 1, learning rate = 0.15

## Discussion

In this study, we utilized a machine learning model to examine acute respiratory tract infections and their determinants among under-five children in Sub-Saharan Africa that can be used for intervention. Among the proposed machine learning algorithms, random forest exhibited superiority with an accuracy of 96.40%, precision of 87.9%, F-measure of 82.8%, ROC curve of 94%, and recall of 78%. Key determinants included maternal age, breastfed, place of delivery, birth size, media exposure, diarrhea, stunting, country, wasting, and weight. The determinants are important for evidence-based decision-making and uncovering hidden patterns in data.

Our results were best with those made in Uganda, which indicated that the random forest model was highly significant for predicting childhood ARI symptoms with an accuracy of 88.70% (33). This might be a result of the disparities in socioeconomic status, culture, way of life, and study area.

By using SHAP values, the findings revealed that having media exposure, being ever vaccinated, giving birth in a health facility, having no diarrhea in the last two weeks, and the normal stunting status were all important variables for the children who had no symptom ARI.

Vaccinated status among children was among the sets of predictors studied in Ethiopia, Tigray regional state, and high mortality counters also support this finding (7, 34, 35). Effective vaccines in childhood prevent key viral respiratory illnesses (36, 37). In the current study, breastfeeding could provide protection against a number of acute gastrointestinal and respiratory illnesses. These findings are supported by similar findings in Ethiopia, Cambodia, Uganda, and Kenya's (3, 33, 38, 39). Due to disparities and barriers to health facilities, it is a big problem that people are less likely to seek healthcare (40). Inadequate facilities may also make it more difficult for mothers to give birth in medical facilities. According to our research, children who were born in medical facilities were more likely to visit medical centers for postnatal care and vaccinations, as well as to seek healthcare overall. Mothers may bring their child for medical attention if they experience any ARI symptoms while traveling to these services. Findings are supported by similar findings (4, 5, 33). Children from rural areas in Sub-Saharan Africa typically get diarrheal diseases as a result of rotavirus infections (41).

Compared to children who have never experienced diarrhea, those who have experienced diarrhea within the last two weeks are more likely to experience ARI symptoms. This is consistent with study findings in Ethiopia (7). ARI was significantly more common in children who were small in birth than in children who were average size, where smaller-sized children had a 18.8% higher chance of developing an ARI. This is consistent with study findings in Ethiopia (42), North Jayapura Sub-District (43). Mothers of a child who had access to media exposure were more likely to seek treatment for ARI (44). Mothers are more likely to seek medical attention when they are exposed to media that alters their views, attitudes, and social norms. This also makes mothers more conscious of the significance and urgency of providing healthcare for their children. The impoverished, however, might not be able to afford radio or television. This study was supported in Bangladesh (45). It has been demonstrated that children who are stunted are more likely to have ARI. This result is also consistent with research carried out in Ethiopia (46, 47). According to this study, children who are malnourished have weakened immune systems and are more vulnerable to ARI and other illnesses.

There were certain restrictions on this investigation. The important variables regarding acute tract infection among under five children due to DHS data collection are self-reported, which may have introduced some information biases. The outcomes of this investigation may also be applied to the development of a mobile application that operates online and anticipates acute respiratory tract infections in children under five. This would enable moms or other caregivers to identify indications of acute tract infection in children who are at high risk early on and help them receive the necessary therapies.

## Conclusion

Using machine learning approaches, it is possible to classify certain secret knowledge that is unable to be classified by conventional statistical tools. Machine learning method approaches have high performance compared to conventional statistical methods. Among the five machine learning models used in this study, the random forest was predicted as the best classifier to be used for the predictive model of acute trace infection and estimating risk factors compared to other machine learning models used in this study. The model of the random forest technique highlighted more important variables, such as revealed ever vaccinate, breastfed, place of delivery, birth size, media exposure, diarrhea, stunting, country, wasting, and weight. It is recommended that policymakers take into account the findings of this research and provide a strategy for prevention of acute trace infection among children in Sub-Saharan Africa based on the relevant variables that have been identified. Even though a fascinating result was obtained, future works were required by applying alternative types of techniques with a different parameter.

### Strength and limitations

This study attempted to forecast acute trace infection and more accurately evaluate the key predictors. Also, this study made use of the DHS data set in Sub-Saharan Africa, which contains almost every demographic risk group that is vulnerable and a large data set. However, this study has certain limitations because the DHS data collection is self-reported, which may have introduced some information biases.

## Data Availability

The datasets presented in this study can be found in online repositories. The names of the repository/repositories and accession number(s) can be found below: http://www.dhsprogram.com.website.
